# Carbamazepine Overdose after Psychiatric Conditions: A Case Study for Postmortem Analysis in Human Bone

**DOI:** 10.3390/toxics10060322

**Published:** 2022-06-13

**Authors:** Lucia Fernández-López, Rosanna Mancini, Maria-Concetta Rotolo, Javier Navarro-Zaragoza, Juan-Pedro Hernández del Rincón, Maria Falcón

**Affiliations:** 1 Department of Pharmacology, Faculty of Medicine, University of Murcia, 30120 Murcia, Spain; lucia.fernandez2@um.es; 2 Instituto Murciano de Investigación Biosanitaria IMIB-Arrixaca, 30120 Murcia, Spain; falcon@um.es; 3 National Centre on Drug Addiction and Doping, Istituto Superior di Sanitá, 00161 Rome, Italy; rosanna.mancini@iss.it (R.M.); mariaconcetta.rotolo@iss.it (M.-C.R.); 4 Forensic and Legal Medicine, Faculty of Medicine, University of Murcia, 30120 Murcia, Spain; jphrincon@um.es

**Keywords:** carbamazepine, bipolar, bone tissue, blood, matrix, concentration

## Abstract

Carbamazepine is the main option used as a preventive medication to treat bipolar disorder when there is no response to lithium. Carbamazepine toxicity is defined as serum levels greater than 12 μg/mL, with severe toxicity occurring over 40 μg/mL, reduced to 30 μg/mL when combined with pharmacological treatment, i.e., benzodiazepines or antidepressants. For these reasons, it is necessary to find a validated tool to determine carbamazepine levels in an autopsy to rule out suicide or to know if the death was a consequence of an adverse drug reaction (ADR), especially when only bones can be accessed. We have validated a tool to detect and quantify drug concentration in bone. Our results showed a peak for carbamazepine at minute 12 and a mass fragment of 193 *m/z*. This case study is the first time in the literature that carbamazepine has been detected and quantified in bone. These results demonstrate that carbamazepine can be detected in bone tissue from forensic cases, but almost more importantly, that the method proposed is valid, reliable, and trustworthy.

## 1. Introduction

Carbamazepine is a dibenzazepine, a drug indicated for use as an antiepileptic drug, but it is also useful to treat other disorders that generate pain, such as trigeminal neuralgia and neuropathic pain and psychiatric conditions including depression and bipolar disorder [1]. Moreover, carbamazepine is the main option used as a preventive medication to treat bipolar disorder when there is no response to lithium [2]. Indeed, it has more effectiveness in avoiding manic episodes than other drugs, with 50% efficacy, and is even more powerful than lithium in preventing relapses [3]. One of the reasons why carbamazepine is not the first choice to treat bipolar disorder is the high rate of toxicity that this drug produces. According to the US Poison Control Center, there are nearly 2000 cases each in the US [4]. Carbamazepine produces a metabolite named oxcarbazepine that has similar effects but fewer adverse drug reactions (ADR) [5]. The recommended plasmatic concentration levels are approximately 4–12 μg/mL with a daily dose of 400 to 1200 mg; meanwhile, carbamazepine toxicity is defined as serum levels greater than 12 μg/mL, with severe toxicity occurring over 40 μg/mL (Table 1) [6,7].

Additionally, patients with combined pharmacological treatment, i.e., benzodiazepines or antidepressants, can generate severe toxicity over 30 μg/mL, affecting the heart and other organs. Furthermore, combination with alcohol can heighten these toxic effects and lead to overdose and, finally, death [6,8] when the use of activated charcoal is not enough [9].

These data, together with the fact that psychiatric patients sometimes have suicidal intentions, show that carbamazepine might be observed and quantified in problematic patients [10]. Nevertheless, when a patient known to be treated with carbamazepine dies, it is necessary to determine the drug levels during the autopsy in order to discard suicide or ADR as a potential cause of death. An autopsy includes post-mortem toxicological analysis to confirm if any drug or toxic has been involved in the death of the person [11]. The matrices usually analyzed are blood, urine, vitreous humor, or pericardial fluid, when available. Some scenes are complicated, and it can be difficult to come to a conclusion about the cause of the death when the body has been buried or hidden for a long time and the matrices are affected [12,13,14]. Sometimes, in post-mortem analysis, blood and urine are not available, so other specimens are submitted, i.e., stomach contents, bile, vitreous humor, liver and other tissues, muscle, or bone marrow [15,16]. In these cases, it can be hard to test and interpret data as these matrices are not frequently analyzed, and therefore there is a lack of literature and published results to compare with [17,18]. The problem becomes larger when the body is fully discomposed, and only bones remain. Toxicological analysis in bones can be performed, although the correlation to blood concentration to complete the post-mortem toxicology is not accessible for carbamazepine, oxcarbazepine, and other similar drugs that have never been quantified in bone. This deficit of data makes it necessary to validate a precise and highly specific method that correlates drug concentration levels in bone to drug concentration levels in blood. The aim of this research was to analyze different post-mortem human rib samples to establish a correlation between carbamazepine levels from bones to blood.

## 2. Materials and Methods

### 2.1. Chemicals and Reagents

We used two internal standards (IS) for carbamazepine and sertraline (Salars, Como, Italy). N,O-Bis(trimethylsilyl)trifluoroacetamide (BSTFA) with trimethylchlorosilane (TFMC) (Sigma-Aldrich, Milano, Italy) is a reagent commonly used in identification and quantification. BSTFA + TMCS has been used as a derivatizing reagent for GC-MS determination of both drugs.

### 2.2. Preparation of Standard Solutions

The standard solutions were prepared according to the following steps. The internal standard solution (100 ng/mL) and the stock solution for the drug studied (1 mg/mL) were stored at 20 °C in methanol. Subsequently, the stock solution was diluted with methanol for the calibration curves and quality control spots. Standards were prepared each day at LOQ values, 5, 50, 100, 250, and 500 ng/mg of the rib by spiking a pre-checked naïve pool with different volumes of methanol. A total of 3 quality control (QC) samples were obtained: 400 ng/mg (high control, QCH), 150 ng/mg (medium control, QCM), and 10 ng/mg (low control, OCL).

### 2.3. Samples

Samples in this research were collected at the Legal Medicine Institute of Murcia, Spain. All the samples were taken from the ribs; specifically, they are the fifth and the sixth ribs’ central parts. This bone was selected because of its vascularity, and it is easily accessed. The length of the bone was 5 cm for all the specimens. Three rib samples from subjects whose blood was free of carbamazepine were collected in order to contrast the data. The institutional Ethical Committee of the University of Murcia approved the study.

### 2.4. Preparation of the Samples and Extraction Procedure

Soft tissues were removed from the surface using a scalpel, and bone samples were chopped into approximately 1 cm fragments using a scalpel and scissors. Samples were dried at 50 °C in an oven overnight and pulverized using a ball mill (Millmix 20, Biogen, Madrid, Spain). The resulting bone powder was restored at −80 °C until the analysis was performed. The analysis of carbamazepine in human bone was carried out according to a validated method [19] with the following steps. A total of 300 mg of bone powder with 1 mg/mL IS solution and 2 mL of methanol were vortexed and incubated for 1 h under ultrasounds. Then, samples were centrifuged, and the supernatants were recovered and evaporated. Phosphate buffered saline (PBS; 0.1 M, pH 6) was added, and samples were subjected to a solid-phase extraction using CleanScreen PKG50 extraction columns (3 cc, 200 mg, United Chemical Technologies, Bristol, PA, USA). Columns were preconditioned with methanol and PBS and samples were loaded. The columns were then washed with deionized water and 0.1 M hydrochloric acid, dried under vacuum, and washed again with methanol. Substances were eluted using 2 mL of dichloromethane:isopropanol:ammonia (78:20:2, *v*/*v*/*v*), and then they were evaporated. Samples were reconstituted with 100 µL of ethyl acetate, vortexed, and pipetted into GC injector vials.

### 2.5. Gas Chromatography–Mass Spectrometry (GC-MS) Analysis

We used a 6890 Series Plus gas chromatograph equipped with an Agilent 7683 autosampler and coupled to a 5973N mass selective detector (Agilent Technologies, Palo Alto, CA, USA) together with a fused silica capillary column (ZB-SemiVolatiles, 30 m, 0.25 mm i.d., 0.25 μm film) from Phenomenex (Torrance, CA, USA). First, 1 µL of the sample was inserted into the injection port held at 260 °C in splitless mode. We maintained the oven temperature at 100 °C for 2 min increasing 30 °C per minute until reaching 190 °C. This temperature was held for 20 min. After this period, we increased 40 °C per minute again. The final selected temperature was 290 °C, which was held for 10 min.

The electron-impact (EI) mass spectra were recorded in total ion monitoring mode (scan range 40–550 *m*/*z*) to determine retention times and characteristic mass fragments of the compounds. Afterward, the instrument was operated in selected-ion-monitoring (SIM) mode. The qualifying ions monitored in SIM mode are displayed in Table 2; the underlined ions were collected and quantified. The ion ratio acceptance criterion was a deviation of ≤20% of the average ion ratios of all the calibrators.

### 2.6. Validation Procedure

Selectivity, carryover, matrix effect, linearity, limits of detection (LOD) and quantification (LOQ), precision, accuracy, recovery, and stability were the parameters calculated in accordance with the criteria described in previous research [20,21,22,23]. Five different daily replicates of the three QC samples were used to calculate validation parameters along three successive working days.

We studied possible interferences by endogenous substances and between carbamazepine and the IS, but also possible carryovers at the drug retention times. Calibration curves were performed in triplicate to analyze linearity. Moreover, peak area ratios between the compound and the IS were quantified. Along with these data, five replicates of blank samples were measured, obtaining a standard deviation (S.D.) of the mean noise level at the retention time window of the compound, which was used for the determination of LOD (3S.D.) and LOQ (10S.D.) of the method. Following this, we analyzed the accuracy and precision of the method at the three QC concentrations, expressed as standard deviation and error (%) of the measured values. Other values such as recovery, matrix effects, and process efficiency were calculated [24]. Finally, we analyzed 3 cycles on QC samples to obtain the mid-term stability of the analytes and their capacity to avoid thaw in bone after freeze (−20 °C). Two samples were quantified in triplicate each month over a period of six months. The stability was expressed as a relative percentage of the initial concentration in QC and in real samples.

### 2.7. Expression of Analyte Levels

Analyte levels were expressed as mass-normalized response ratios (RR/m), RR for the ratio between peak areas of the ion and the internal standard. This method allows valid results when [14,21,22,23] it is not possible to have analyte recoveries from bone tissue that provide accurate data. RR/m values were expressed in concentration units (ng/mg).

## 3. Results

### 3.1. GC-MS

**Figure 1:**
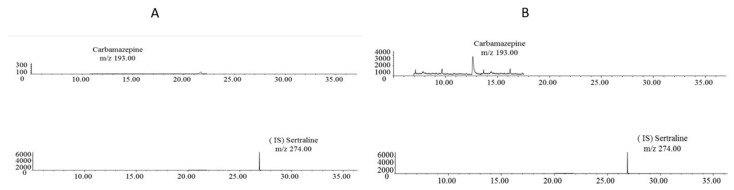
(**A**) Representative chromatograms obtained following the extraction of 0.3 g of drug-free bone pool. (**B**) SIM chromatogram of an extract of 0.3 g of drug-free bone pool spiked with 50 ng of carbamazepine.

The chromatogram of the drug-free bone pool samples did not show any background of endogenous substances once the extraction was performed (Figure 1A). This chromatogram was certified as representative. Furthermore, in Figure 1B, a representative chromatogram for an extract of 0.3 g spiked with 50 ng of carbamazepine. The peak for carbamazepine usually is set at minute 12, and its mass fragment is 193 *m/z*. We did not see traces of carryover after the calibration curve’s highest point in any controlled sample.

### 3.2. Validation Results

The determination coefficient (r^2^) for the linear calibration curve was 0.999 up to 500 ng/mg in the sample studied. We obtained a LOD smaller than 0.1 ng/mg and a LOQ of 0.3 ng/mg. The precision obtained intra- and inter-day was not bigger than 2.5% for all the collected data injected, which assure repeatability. We also repeatedly obtained a result of over 14% for the inter-and intra-assay accuracy values (Table 3). We found an analytical recovery of 92.6%, which confirms a proper extraction activity. The matrix effect was also calculated, resulting in 78.8%. Because of this, the matrix did not significantly change the intensity of the signal. Regarding efficiency, our results showed 73%. We also saw that the QC samples were stable during all the cycles (3 freeze/thaw). We had differences in concentration under 10% if we compare with the value at time 0. Regarding the stability of the samples at mid-term, we also noted a difference below 10%. This allows the samples to be stored until the analysis in the different institutes of legal medicine.

### 3.3. Application to Real Samples

**Figure 2:**
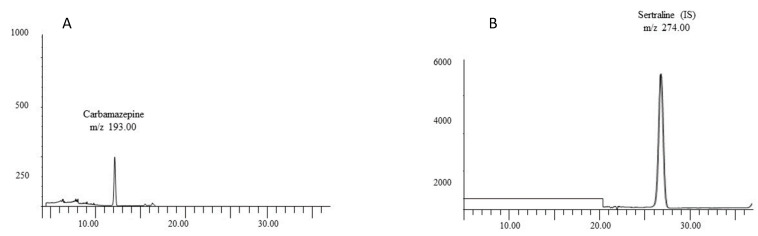
(**A**) SIM chromatogram of bone extracts from the real case containing approximately 46 ng/mg bone of carbamazepine. (**B**) SIM chromatogram for sertraline (IS) of bone extracts from the real case.

Once the method was tested and validated, we performed it to see the results in a forensic case with a blood-positive result for carbamazepine. The sample was obtained from a Caucasian male aged 41-years-old who probably died due to chronic ischemic heart disease and had a post-mortem interval of approximately 13 h. The result for the concentration of carbamazepine in blood was 3750 ng/mL, which is within the therapeutic blood range [25]. It was also detected in the bone sample at 46 ng/mg. A representative chromatogram after this extraction is shown in Figure 2.

## 4. Discussion

This is the first time in the literature that carbamazepine was detected in bone; therefore, these results demonstrate that carbamazepine may be detected in bone tissue in forensic cases. We have previously validated a method to detect different drugs in bones. The method has been tested in this case study and seems to be valid, reliable, and trustworthy, although more studies should be performed. A procedure that includes the detection of this drug in bone is interesting since it is an increasingly used substance prescribed for multiple therapeutic indications, but especially for psychiatric diseases. Carbamazepine is used in some cases by patients suffering from schizophrenia, bipolar disorder, mania, or major depression, which are disorders characterized by unstable behaviors, abrupt changes in mood, or hallucinations in some cases. These situations may lead to cases of medico-legal interest such as suicides, aggressions, or murders since these mental disorders have been associated with the risk of premature death from suicide and other causes [26], violence, and violent offending, particularly homicide [27], and repeat incarcerations [28].

Postmortem toxicological results in bone are hard to understand since there is no standardized data about toxic, therapeutic, or even lethal concentrations of drugs in bone. It is important to clarify that quantitative data about drugs in bone would not give more information than qualitative data. If a database were developed with all these parameters, this interpretation would be much easier. The problem is that the mentioned database exists only for blood matrix, so the correlation of drug levels between blood and bone would also give interpretative value to quantitative data in bone.

## 5. Conclusions

This method for the detection of carbamazepine in human bone was tested and validated, providing satisfactory results in a real forensic case. Although there is a huge lack of results in bone matrix, detection of the different drugs could help to establish a correlation between results in blood and bone, allowing forensics to know the cause of death even when the victims have been buried or have been disappeared for a long period of time. We encourage other researchers to expand the number of substances detected in bone in order to make this matrix a valid tool for every case in the future.

## Figures and Tables

**Table 1:** Carbamazepine indications, pharmacokinetics, and reference blood levels.

Indication	Pharmacokinetic	Blood Levels
Main indication: epilepsy Other indications: trigeminal neuralgia neuropathic pain psychiatric conditions (depression, bipolar disorder, manic episodes)	Distribution: 70–80% protein binding Reaches breast milk and crosses placental barrier. Metabolism: hepatic Elimination: 70% renal, 30% hepatic t_1/2_ 36 h	Terapeutic: 4–12 μg/mL Toxic: >12 μg/mL Severe toxicity: 40 μg/mL (in combination with antidepressants or alcohol: 30 μg/mL)

**Table 2:** Retention times and characteristic ions of analyzed substances by GC-MS.

Substance	RT (min)	Characteristic Mass Fragments (*m/z*)
Carbamazepine	12.9	193–165–139
Sertraline (IS)	26.8	159–262–274–304

**Table 3:** Intra- and inter-assay (*n* = 3) precision and accuracy obtained for carbamazepine.

Analyte	Intra-Assay Precision (RSD)			Intra-Assay Accuracy (ABS%Error)			Inter-Assay Precision (RSD)			Inter-Assay Accuracy (ABS%Error)		
	* QCL	** QCM	*** QCH	QCL	QCM	QCH	QCL	QCM	QCH	QCL	QCM	QCH
Carbamazepine	1.4	2.3	0.4	13.6	3.3	1.9	1.3	1.4	0.9	6.9	0.2	1.0

* QCL: 10 ng/mg. ** QCM: 150 ng/mg. *** QCH: 400 ng/mg.

## Data Availability

Data available on request due to restrictions.
